# DAER-YOLO: Defect-Aware and Edge-Reconstruction Enhanced YOLO for Surface Defect Detection of Varistors

**DOI:** 10.3390/jimaging12050198

**Published:** 2026-05-02

**Authors:** Wu Xie, Shushuo Yao, Tao Zhang, Gaoxue Qiu, Dong Li, Fuxian Luo, Yong Fan

**Affiliations:** 1 School of Computer Science and Information Security, Guilin University of Electronic Technology, Guilin 541004, China; 14769750797@163.com; 2 Guangxi Key Laboratory of Trusted Software, Guilin University of Electronic Technology, Guilin 541004, China; 3 Guangxi Lipu Yimeida Household Products Co., Ltd., Heanke Village, Maling Town, Lipu City, Guilin 546600, China; 4 Guilin Maojia Crafts Co., Ltd., Huangzhai Industrial Zone, Licheng Town, Lipu City, Guilin 546600, China; 5 School of Mechanical and Electrical Engineering, Guilin University of Electronic Technology, Guilin 541004, China; fywsq@guet.edu.cn

**Keywords:** defect detecting, deep learning, attention mechanism, YOLO

## Abstract

Varistors are critical overvoltage protection components in modern power electronic systems. They effectively absorb and dissipate surge energy to ensure the safe and stable operation of electrical equipment. However, surface defects can lead to substandard performance or even trigger equipment failure, compromising overall system stability. Therefore, high-precision surface defect detection is essential for quality assurance. To address these challenges, we propose a lightweight model termed Defect-Aware and Edge-Reconstruction Enhanced YOLO (DAER-YOLO) for efficient varistor inspection. First, we construct a C3k2-based defect-aware enhancement module (C3k2-iEMA). This module tackles the difficulty of extracting features from small or morphologically complex defects. By integrating multi-scale feature extraction, an attention mechanism, and efficient nonlinear mapping, it strengthens the perception of defect details. Second, to enhance the reconstruction capability for edge damage and small-object defects, we introduce the Efficient Up-Convolution Block (EUCB). This block improves multi-level feature fusion and generates clearer enhanced feature maps. Based on these improvements, DAER-YOLO outperforms the YOLOv11n baseline on a custom varistor dataset, with mAP@50 and mAP@50:95 increasing by 1.6% and 2.3%, respectively. Experimental results demonstrate that the model effectively improves detection accuracy while exhibiting significant potential for real-time industrial applications.

## 1. Introduction

A varistor is a clamping-type device that suppresses transient overvoltage through its nonlinear characteristics to protect downstream circuits [1,2] ADDIN EN.CITE. It serves as a core component for ensuring the safe and stable operation of electrical equipment. However, limitations in manufacturing equipment precision, material homogeneity, and environmental factors inevitably introduce surface defects during production. Typical defects include poor coating, surface contamination, and edge damage. These defects cause the electrical characteristics of varistors to deviate from design specifications and can trigger failures under high-voltage conditions. Consequently, they severely compromise overall system reliability and service life. Therefore, the efficient and accurate inspection of varistor surface defects is critical for ensuring product quality.

With the rapid advancement of computer vision and deep learning, surface defect inspection has gradually shifted from traditional machine vision and unsupervised reconstruction to end-to-end deep learning frameworks [3]. Unsupervised reconstruction methods typically rely exclusively on defect-free samples for training. By learning the texture distribution of normal patterns, these models reconstruct input images into “defect-free” versions during inference. They then localize defects based on the residual differences between the original and reconstructed images [4]. Although advantageous when defective samples are scarce, these methods often exhibit limited sensitivity to subtle, low-contrast, or edge-blurred defects. Moreover, over-reconstruction can suppress critical defect cues, thereby restricting the model’s overall robustness and generalization. In contrast, Convolutional Neural Network (CNN)-based detectors [5], such as Faster R-CNN [6], the YOLO family [7], and RetinaNet [8], have markedly improved detection accuracy and automation in industrial applications. The YOLO series, in particular, leverages an end-to-end single-stage architecture to achieve excellent real-time performance in manufacturing settings. However, detecting defects on small electronic components like varistors remains challenging. These surface defects are typically tiny, morphologically complex, and highly susceptible to specular reflections. Consequently, existing deep learning models often struggle with small-object recognition accuracy, edge-detail restoration, and sufficient feature representation. Although Transformer-based architectures [9] provide strong global modeling capabilities, their high computational costs and difficult training convergence limit their deployment on production lines. Furthermore, varistor inspection systems typically run on embedded industrial vision devices or edge-computing terminals. These devices impose strict constraints on computation, memory, and power budgets, making real-time inference with large models impractical. Therefore, the industry urgently needs a lightweight detection model that maintains high accuracy under limited resources, perceives small defects with high sensitivity, and supports real-time industrial deployment.

Accordingly, we propose a lightweight object detection model termed Defect-Aware and Edge-Reconstruction Enhanced YOLO (DAER-YOLO). Built upon YOLOv11n [10,11], this model specifically addresses the challenges of small-scale defects, complex patterns, and blurred edge features on varistor surfaces. To achieve this, we introduce two key mechanisms. First, we design a feature enhancement module (C3k2-iEMA) that integrates an Efficient Multi-scale Attention (EMA) mechanism [12] with an inverted residual structure (iRMB) [13]. This module significantly improves fine-grained defect feature representation and perception. Second, we construct an Efficient Up-Convolution Block (EUCB) [14] to optimize the feature reconstruction process. The EUCB strengthens cross-level feature fusion and enhances the reconstruction quality of defective boundaries. Within the DAER-YOLO framework, these modules operate collaboratively in an end-to-end manner. They effectively improve detection accuracy and robustness for small targets and complex defects while maintaining high computational efficiency. Consequently, our proposed model provides a highly practical technical solution for inspecting electronic components characterized by complex edges and small-scale flaws.

The main contributions of this study are summarized as follows:

1. We constructed a real-world varistor surface defect dataset to meet the practical requirements of industrial manufacturing. We acquired the image data using an industrial camera and a precision 3D acquisition platform. The dataset encompasses eight typical defect categories (e.g., pattern misalignment, poor coating, pinhole, and edge damage). We carefully annotated and partitioned the data based on explicit classification criteria. Furthermore, we applied data augmentation strategies to expand the sample size and enrich distribution diversity. This provides robust and reliable data support for model training and performance evaluation.
2. We propose a novel defect-aware enhancement module, termed C3k2-iEMA. Existing attention-based models often struggle to balance global context with local details. In contrast, our proposed C3k2-iEMA uniquely integrates multi-scale feature extraction (EMA) with an inverted residual structure (iRMB). This captures diffuse, low-contrast features without the high computational overhead typical of Transformer models.
3. We introduce an edge-reconstruction optimization mechanism by adopting the Efficient Up-Convolution Block (EUCB). Conventional lightweight detectors rely on standard upsampling operators, which frequently blur irregular boundaries. To solve this, we integrate EUCB to facilitate cross-channel information interaction during feature reconstruction. The synergistic combination of C3k2-iEMA (for defect perception) and EUCB (for edge preservation) sets DAER-YOLO apart from existing methods, successfully resolving the feature loss bottleneck for edge damage and tiny defects in industrial varistor inspection.

## 2. Related Work

Early studies on varistor surface defect inspection primarily relied on traditional machine vision pipelines. These methods performed defect recognition using image preprocessing and classical machine learning models. For instance, researchers combined algorithms like K-means clustering [15], decision trees [16], and Support Vector Machines (SVM) [17] with handcrafted descriptors—such as gray-level co-occurrence matrices, edge contours, and texture features—to classify surface defects. Although widely used in early academic research, these approaches rely heavily on manual feature engineering. Consequently, the quality of the handcrafted features fundamentally constrains their performance, resulting in limited robustness and weak generalization. The advancement of Convolutional Neural Networks (CNNs) [5,6] shifted defect detection toward an end-to-end deep learning paradigm. Modern deep learning models automatically extract multi-scale and semantically rich feature representations. This paradigm shift has substantially improved both detection accuracy and system adaptability [18].

In recent years, researchers have actively explored network architecture refinement and feature enhancement for varistor defect inspection. For example, Peng et al. [19] developed the VGG-8-LRN model by streamlining the convolutional layers of VGG-16 and introducing Local Response Normalization (LRN), which improved both the sensitivity and specificity of defect detection. To address the scarcity of real defective samples, Chen et al. [20] constructed a CNN-based framework and employed Generative Adversarial Networks (GANs) to synthesize high-quality training images. This strategy effectively alleviated the performance degradation caused by limited data.

Unsupervised and generative approaches have also shown promise. Chen et al. [21] proposed a spatial-attention-enhanced Deep Convolutional Variational Autoencoder (DCVAE). Their model reconstructs defect-free images by encoding the features of normal samples and localizes defects using residual maps while suppressing background noise. Similarly, Tang et al. [22] presented a deep transfer DCVAE network to enable adaptive image preprocessing and defect detection across different product variants. More recently, Tang et al. [4] proposed an unsupervised method based on normal distribution reconstruction and mask constraints. By integrating KAN, U-Net, and Gaussian Radial Basis Functions (GRBFs) alongside a pseudo-defect generation strategy, their model effectively enhances high-dimensional feature representation and precisely localizes tiny defects in multi-specification varistors.

In the realm of lightweight Object Detection, Tang et al. [23] integrated depthwise separable modules and attention mechanisms (channel and spatial) into YOLOv3-Tiny. This modification strengthened feature extraction and significantly reduced false positives and missed detections. Finally, Wu et al. [24] introduced a highly optimized scheme based on YOLOv5s by combining knowledge distillation, channel pruning, and post-training quantization (PTQ). They designed an edge-cloud collaborative solution that jointly performs binary classification and object detection, drastically reducing computational overhead while maintaining stable performance.

Although recent studies have advanced varistor surface defect inspection, existing methods still exhibit clear limitations in complex industrial scenarios. First, current detectors lack sufficient feature representation capabilities to handle small defects, specular reflections, and highly irregular defect shapes. This deficiency often leads to suboptimal accuracy and instability. Second, conventional feature reconstruction processes easily lose critical details for edge damage and minute flaws, resulting in localization bias and missed detections. To address these critical issues, we propose DAER-YOLO, a novel model that integrates defect-aware perception with an edge-reconstruction enhancement mechanism. Furthermore, we optimize this model using targeted training strategies and data augmentation. Comparative experiments demonstrate that DAER-YOLO outperforms existing approaches in overall detection accuracy, effectively recognizing small-scale defects and complex morphological patterns.

## 3. Materials and Methods

### 3.1. Data Acquisition and Analysis

**Figure 1:**
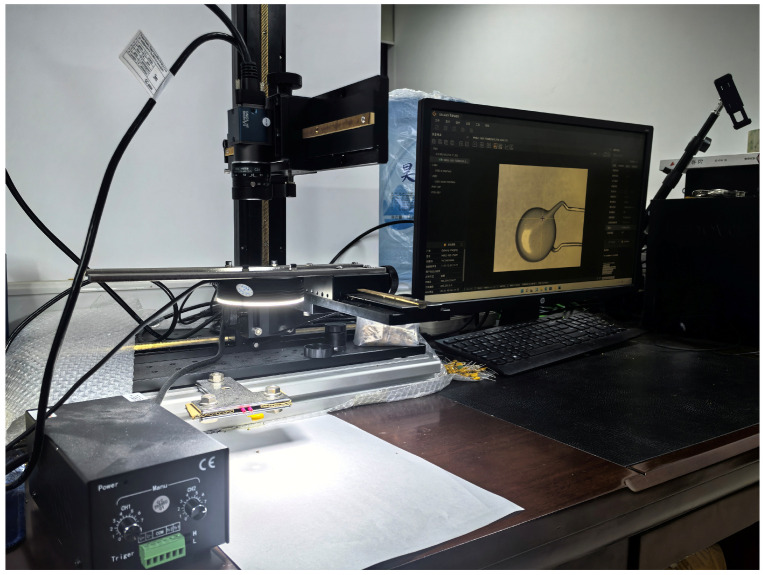
Photograph of the 3D precision translation acquisition platform.

Systematic image acquisition and analysis form the foundation for constructing a high-quality deep learning dataset. We first investigated defect characteristics, defined classification criteria, and assessed environmental impacts. These steps establish a robust basis for subsequent system design and algorithm optimization. For this study, an electronic component manufacturer in Guangxi, China, provided the defective varistor samples. We captured and curated the dataset images using a custom-developed acquisition platform. The collected data encompasses eight common surface defect categories: pattern misalignment, poor coating, surface contamination, pinhole, pitting, edge damage, missing bottom, and contour deformation. To preserve fine-grained textures and edge details, we acquired all images at a uniform resolution of 800 × 654 pixels and stored them in the lossless Bitmap (BMP) format. Furthermore, to ensure multi-view coverage, high precision, and an accurate representation of real-world inspection environments, we conducted image acquisition using a 3D precision platform equipped with an industrial camera and a ring-shaped LED light source. Figure 1 illustrates this 3D precision translation platform.

**Figure 2:**
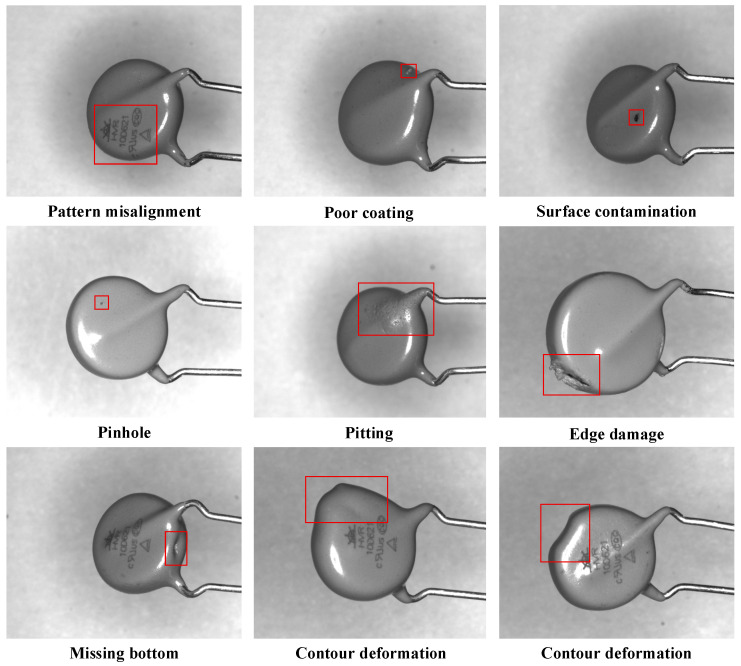
Representative examples of the eight typical surface defect categories on varistors.

During image acquisition, we tilted the varistor samples by 15° to 30° on the precision platform to ensure clear imaging of defect features in both the top and bottom regions. After data collection and rigorous image screening, we retained a total of 1919 high-quality images. To facilitate model training and evaluation, we randomly partitioned the dataset into training, validation, and test sets at a ratio of 7:2:1. Specifically, this split yields 1343 images for training, 384 for validation, and 192 for testing. Finally, Figure 2 displays representative examples of the eight typical defect categories within our dataset.

**Figure 3:**
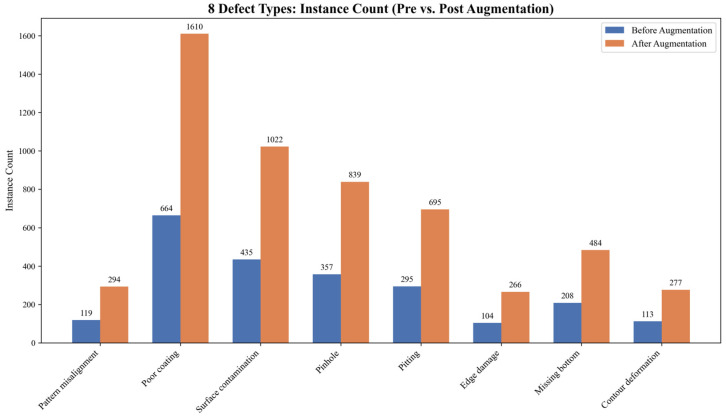
Bar chart of instance counts for various defect categories before and after augmentation.

In object detection tasks, insufficient training samples often cause models to overfit, thereby reducing their generalization ability [25]. To ensure stable detection performance in complex industrial environments, we applied offline data augmentation to the original training set. Specifically, we employed strategies such as mirror flipping, random rotation, and random scaling [26]. These techniques tripled the total number of training images, expanding the set to 4029 samples. Figure 3 compares the instance counts of the eight typical defect categories before and after data augmentation. The statistical results reveal a clear long-tail distribution within the dataset. For example, poor coating and surface contamination occur frequently, whereas edge damage and pattern misalignment are relatively rare. Because this long-tail distribution accurately reflects the natural occurrence of defects in varistor production, maintaining the original data distribution helps the model learn the true prior probabilities of defects. Consequently, this strategy significantly reduces the risk of false detections during real-world deployment.

Furthermore, to quantify the difficulty of detecting small defects, we analyzed the pixel area distribution of the ground truth bounding boxes in our dataset. Following the standard MS COCO evaluation criteria, we define small targets as those with an area smaller than 32 × 32 pixels. Out of 2295 total defect instances in the dataset, 1189 (51.81%) qualify as small targets. This high proportion of small targets indicates that conventional lightweight models are highly susceptible to feature loss and missed detections in such scenarios. Consequently, these statistical results provide objective data support for our proposed network design. Specifically, during the feature extraction and fusion stages, the architecture must incorporate mechanisms that enhance fine-grained perception and preserve local features to effectively mitigate the missed detection of tiny defects.

### 3.2. Methods

In this section, we propose the DAER-YOLO method to address the challenges of detecting small-object defects and edge damage on varistor surfaces. This method integrates defect perception enhancement with edge reconstruction optimization. Specifically, at the feature representation level, we introduce the improved C3k2-iEMA module to enhance the model’s perception of small targets and low-contrast defects. At the feature reconstruction level, we incorporate the EUCB upsampling mechanism [14] to strictly preserve edge details and improve contour reconstruction quality. By synergizing these two core improvements, we construct the complete DAER-YOLO network architecture.

#### 3.2.1. DAER-YOLO Network Architecture

**Figure 4:**
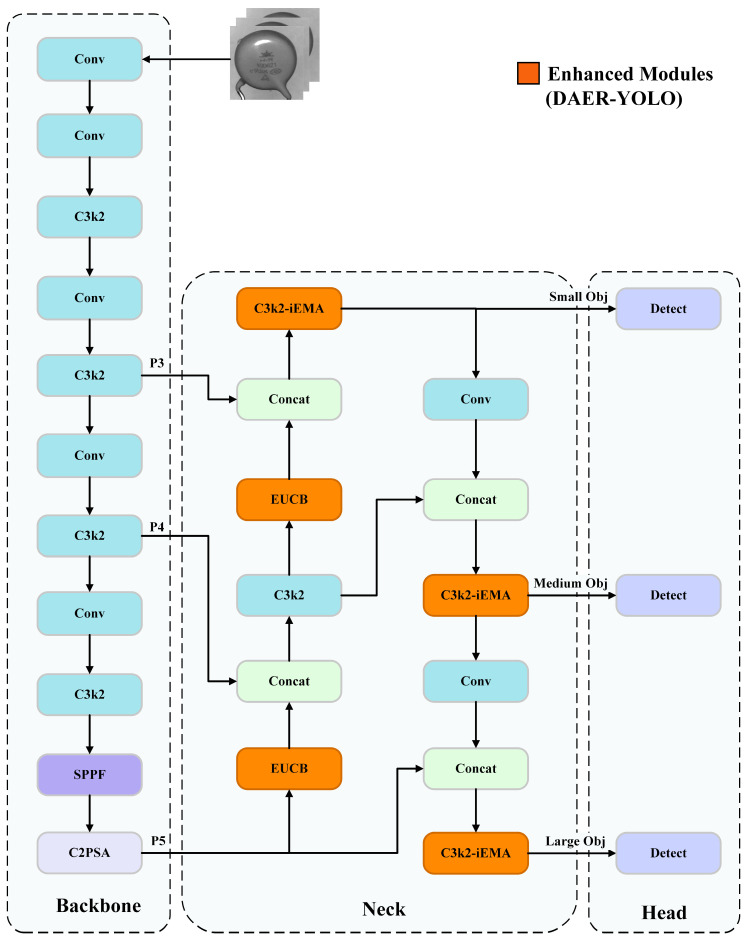
Overall architecture of DAER-YOLO, where the orange-colored modules represent the proposed improvements.

The proposed DAER-YOLO model builds upon the YOLOv11n baseline network [10,11]. Figure 4 illustrates its overall architecture, where the orange modules highlight our proposed architectural improvements.

To preserve the lightweight nature of the model, DAER-YOLO retains the backbone and detection head of the original YOLOv11n. Instead, we concentrate all architectural modifications within the Neck feature fusion network. To combat feature degradation in complex defect scenarios, we introduce two key structural redesigns to the Neck: the C3k2-iEMA feature enhancement module and the EUCB upsampling module.

First, we design and embed the C3k2-iEMA composite module at critical feature fusion nodes. Features of small-object defects are highly prone to degradation during multi-scale propagation. To mitigate this, we replace several standard C3k2 modules in the Neck network with our proposed C3k2-iEMA. This new module retains the original cross-stage partial network topology but cascades an iEMA mechanism and a Feed-Forward Network (FFN) [27] at its output. During the fusion of low-level spatial details with high-level semantic information, this architectural enhancement significantly strengthens the network’s focus on low-contrast regions. Consequently, it effectively boosts the overall detection rate for small-object defects.

Second, we introduce the Efficient Up-Convolution Block (EUCB) into the upsampling path. During the top-down feature fusion stage of the original YOLOv11n, standard upsampling operators often cause feature blurring when processing defects such as edge damage and contour deformation. To resolve this issue, we replace the standard upsampling operators in the Neck network with EUCB modules. This module reconstructs the upsampling process using Depthwise Separable Convolutions (DWConv) and pointwise convolutions. Consequently, it assigns higher network weights to boundaries and complex textures. This optimizes the edge reconstruction of defect contours without significantly increasing computational overhead.

#### 3.2.2. C3k2-iEMA Module

**Figure 5:**
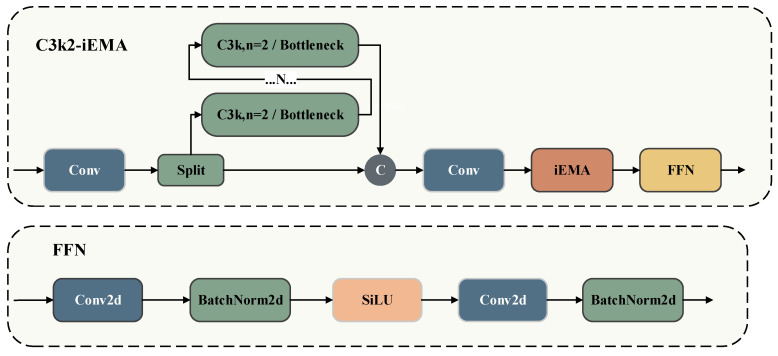
Network architecture of C3k2-iEMA and FFN.

In varistor surface defect inspection, identifying small-object defects and accurately characterizing complex morphological features are crucial. To achieve this, we design a defect-aware module, termed iEMA, by leveraging the Efficient Multi-scale Attention (EMA) mechanism [12] and the inverted residual structure (iRMB) [13]. We then integrate this iEMA module into the C3k2 architecture to enhance the network’s feature representation and defect perception capabilities. Figure 5 illustrates the complete architecture of the C3k2-iEMA module.

The C3k2-iEMA module upgrades the original C3k2 structure by incorporating an iEMA module and a Feed-Forward Network (FFN) [27] layer. Within the Neck network, this integration significantly strengthens the model’s multi-scale perception of varistor defects. Specifically, appending the FFN immediately after the iEMA module improves the non-linear transformation of the features, thereby enriching their overall representational capacity.

**Figure 6:**
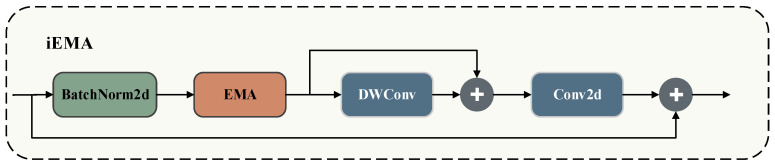
Architecture of the iEMA module.

The iEMA module is designed to balance attentional focus with efficient feature transformation. The standard EMA mechanism effectively fuses multi-scale features and highlights defect regions using parallel subnetworks and cross-spatial learning; however, its feature transformation capacity remains inherently limited. Conversely, the iRMB structure expands channel dimensions and employs Depthwise Separable Convolutions (DWConv) to strengthen feature extraction, yet it lacks the ability to selectively focus on critical regions. To bridge this gap, we embed the EMA mechanism directly into the iRMB backbone. This integration enables the iEMA module to adaptively focus on defect regions during feature extraction. Furthermore, it enhances multi-scale defect perception. By leveraging the efficient design of iRMB, it minimizes computational overhead, achieving an optimal balance between accuracy and efficiency. Figure 6 illustrates the detailed architecture of the iEMA module.

Within the iEMA module, the input feature map $F\in {\mathbb{R}}^{C\times H\times W}$ first undergoes Batch Normalization (BN) before entering the EMA unit. By fusing multi-scale features and modeling long-range dependencies, the EMA unit strengthens the overall representational power and captures essential semantic information across varying scales. Next, the integrated iRMB structure utilizes Depthwise Separable Convolutions (DWConv) and pointwise convolutions for highly efficient feature extraction. This streamlined design preserves robust feature representations while significantly reducing computational complexity. Finally, Equations (1)–(3) formalize the exact computational workflow of the iEMA module:

(1) $$F_{0}=EMA(BN(F))$$

(2) $$F_{1}=F_{0}+DWC_{3\times 3}(F_{0})$$

(3) $$F_{out}=\phi_{1\times 1}(F_{1})+F$$

where $F_{t}\in {\mathbb{R}}^{C\times H\times W},t\in \left\{0,1,out\right\}$ denotes the feature map at scale *t*; *BN* represents Batch Normalization; *EMA* stands for the Efficient Multi-scale Attention module, which is utilized to enhance feature representations; $DWC_{3\times 3}$ denotes a depthwise separable convolution with a 3 × 3 kernel; and $\phi_{1\times 1}$ represents a 1 × 1 pointwise convolution primarily used for channel compression and feature fusion. Ultimately, these operations yield the final output $F_{\mathrm{out}}$ a defect-aware enhanced feature representation that significantly boosts the saliency and discriminability of the target regions.

**Figure 7:**
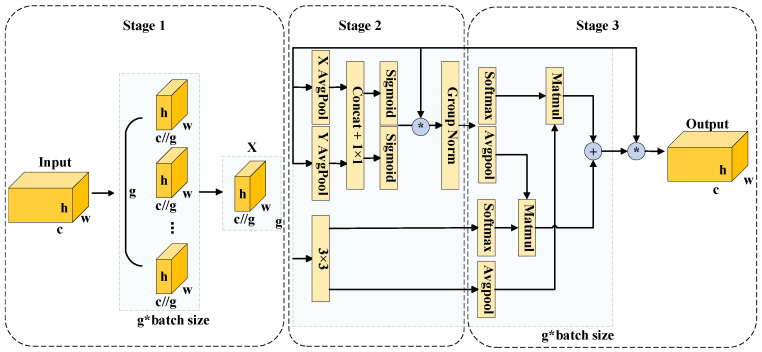
Architecture of the EMA module. The * symbol in the figure (within the circle) denotes element-wise multiplication. It represents a learning paradigm that fuses features from different subspaces via element-wise multiplication.

Figure 7 illustrates the three-stage architecture of the EMA module: feature grouping, parallel sub-networks, and cross-spatial learning. During the feature grouping stage, given an input defect feature map $X\in {\mathbb{R}}^{C\times H\times W}$, the EMA mechanism splits X into G sub-feature groups along the channel dimension to model hierarchical semantic information independently. This grouping strategy effectively reduces computational complexity and parameter overhead while enriching the diversity of feature representations. Consequently, it captures fine-grained defect characteristics more comprehensively across multi-scale and multi-semantic spaces, establishing a robust foundation for the subsequent parallel modeling and cross-spatial learning stages. Finally, Equation (4) formalizes this grouping operation:

(4) $$X=\left[X_{0},X_{i},\ldots ,X_{G-1}\right],X_{i}\in {\mathbb{R}}^{C//G\times H\times W}$$

Following feature grouping, parallel sub-networks further process each sub-feature group. Each sub-network comprises three parallel paths and a residual connection designed for highly efficient cross-spatial feature fusion. Specifically, the first two paths utilize 1 × 1 convolutions to encode channel information along the horizontal and vertical directions, respectively. We spatially concatenate these encoded features, fuse them using an additional 1 × 1 convolution, and split them to generate enhanced horizontal and vertical feature maps. Next, we normalize these features using a Sigmoid activation function and apply element-wise multiplication with the original grouped features. Integrating this output with a residual connection and Group Normalization (GN) significantly elevates the representational capacity of the channel features, yielding defect feature maps enriched with directional attention. Meanwhile, the third path employs a 3 × 3 convolution for local feature modeling. This operation effectively captures multi-scale defect patterns and provides a richer feature representation for the subsequent cross-spatial learning stage. Through this architectural design, the parallel sub-networks achieve differentiated modeling across both channel and spatial dimensions. By seamlessly balancing local and global features, they substantially enhance the model’s perception capabilities and robustness when inspecting complex defect regions.

Following feature extraction in the parallel sub-networks, the EMA module enters the cross-spatial learning stage. This phase aims to fuse feature information from various groups and paths, facilitating the synergistic modeling of global context and local details. Specifically, the grouped directional attention features and multi-scale convolutional features undergo Global Average Pooling (GAP) and Sigmoid activation. We then execute element-wise weighting and feature interaction across the spatial dimension to achieve efficient cross-spatial fusion. Furthermore, residual connections preserve the original input information, while non-linear activation and normalization further enhance the discriminative power of the features. The resulting feature maps effectively balance global consistency with local defect details, enabling the precise localization of varistor surface defects. By incorporating cross-spatial learning, the EMA module performs fine-grained learning within groups and facilitates contextual interactions between them. This significantly improves the network’s representation and generalization for complex defects.

#### 3.2.3. EUCB Module

**Figure 8:**
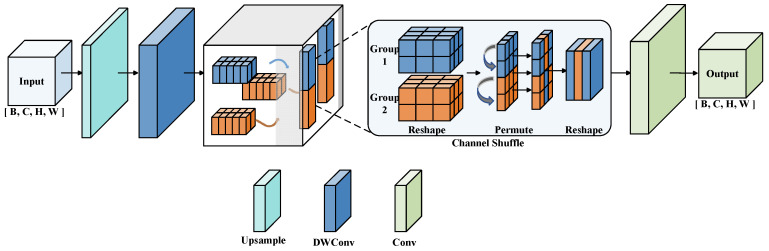
Architecture of the EUCB module.

During the feature reconstruction stage, conventional upsampling operations frequently result in the loss or blurring of edge information, particularly when handling irregular edge damage and contour deformation on varistor surfaces. To enhance the restoration of such defects, we incorporate the Efficient Up-sampling Convolution Block (EUCB) [14] to replace the standard upsampling layer in the baseline model. Figure 8 illustrates the architecture of the EUCB module, which comprises upsampling, Depthwise Separable Convolution (DWConv), channel shuffle, and pointwise convolution. The core design philosophy of EUCB is to effectively preserve high-frequency edge details. It achieves this by leveraging channel shuffle and DWConv, while maintaining a lightweight computational footprint. For varistor inspection, this mechanism specifically aligns with the physical characteristics of edge damage, which is typically irregular and highly susceptible to reconstruction blurring.

First, the module employs an upsampling operation to restore feature map resolution, mitigating the information loss caused by previous downsampling. Subsequently, Depthwise Separable Convolution (DWConv) extracts local spatial features while minimizing computational cost, thereby increasing the model’s sensitivity to edge damage, missing bottom, and small-object defects. To further refine feature representation, EUCB incorporates a channel shuffle mechanism to break channel independence and facilitate cross-channel information interaction. Finally, a 1 × 1 pointwise convolution integrates and reconstructs the feature distribution, ensuring that the upsampled features retain both rich local details and global consistency. Beyond high-quality edge reconstruction, EUCB effectively recovers the features of small-object defects. Due to its lightweight and efficient design, the integration of EUCB enhances the model’s ability to preserve edge information and improves the localization accuracy of minute defect regions, ultimately leading to superior overall detection performance.

#### 3.2.4. Varistor Surface Defect Detection Method Based on DAER-YOLO

Algorithm 1 details the inference pipeline of our DAER-YOLO-based varistor surface defect detection method. The process begins with a varistor surface image containing multiple defect types (e.g., pattern misalignment, poor coating, surface contamination, and pinhole).

First, the backbone network extracts multi-scale semantic feature maps from the input image. Next, the Neck network incorporates the C3k2-iEMA module to fuse multi-scale convolutional features with an efficient attention mechanism, thereby enhancing the model’s perception of small objects and complex defective regions. Subsequently, the EUCB performs cross-level feature fusion and edge reconstruction, effectively mitigating feature loss for tiny defects during the upsampling process.

The detection head then predicts classification scores and bounding-box offsets; specifically, the classification branch estimates defect category probabilities, while the regression branch decodes grid coordinates to generate candidate detection boxes. Finally, Non-Maximum Suppression (NMS) filters redundant predictions to output the final defect categories, locations, and confidence scores. This pipeline enables the precise detection and visual localization of diverse defect types on varistor surfaces.
**Algorithm 1** Inference Algorithm for surface defect detection of varistors based on DAER-YOLO
***Input****: An input varistor surface image Ic with shape H* × *W* × 3. ***Output****: Predicted class labels, bounding boxes, and confidence scores* *1: multi_scale_features = Backbone(Ic)*
*2: enhanced_feats = []*
*3: **for each** feature_map **in** multi_scale_feats **do**:*
*4:      feat_iema = C3k2_iEMA(feature_map)* *5:      enhanced_feats.append(feat_iema)* *6: **End for*** *7: fused_feats = Neck_Fusion(enhanced_feats)* *8: refined_feats = EUCB(fused_feats)* *9: **for each** detection_head **in** refined_feats **do**:* *10:      cls_feat, reg_feat = Split(det_head)* *11:      pred_scores = ClassConv(cls_feat)* *12:      bbox_preds = BoxConv(reg_feat)*
*13: **End for*** *14: decoded_boxes = DecodeBBox(bbox_preds, anchors)*
*15: results = NMS(decoded_boxes, pred_scores, iou_threshold)* *16: **return** results.classes, results.boxes, results.scores*

## 4. Results

### 4.1. Experimental Environment and Settings

We implemented and evaluated the proposed model using the PyTorch 2.0.1 deep learning framework. The hardware configuration consisted of an 11th Gen Intel(R) Core(TM) i7-11700K CPU @ 3.60 GHz and an NVIDIA Quadro RTX 5000 GPU with 16 GB of VRAM. The experiments were conducted on a Windows 10 operating system, with a software environment comprising Python 3.11.9, CUDA 12.4, and cuDNN 8.9.7. During training, we set the input image resolution to 640 × 640 pixels, with a batch size of 16 and 8 data loader workers. We employed the Stochastic Gradient Descent (SGD) optimizer with an initial learning rate of 0.01, a weight decay of 0.0005, and a momentum factor of 0.937. The network was trained for a total of 400 epochs. Notably, we deactivated the mosaic data augmentation strategy during the final 10 epochs to stabilize convergence and enhance the model’s generalization performance.

To ensure objectivity and fairness in our comparative analysis, we trained all baseline models from scratch using identical hardware/software environments and dataset partitions. Specifically, to allow each model to reach its peak baseline performance, we applied the official default hyperparameters for the DINO model, while the YOLO series and RT-DETR followed the default optimal configurations within the YOLOv11 framework. Furthermore, we clarify key implementation details to guarantee the reproducibility of our results. Since the baseline YOLOv11n employs a modern anchor-free mechanism, we eliminated the need for manual anchor box clustering. Regarding the loss functions, we utilized Binary Cross-Entropy (BCE) for classification loss and a combination of Complete IoU (CIoU) and Distribution Focal Loss (DFL) for bounding box regression loss. During the inference and evaluation phases, we consistently set the confidence threshold to 0.25 and the IoU threshold for Non-Maximum Suppression (NMS) to 0.45.

### 4.2. Evaluation Metrics

To comprehensively and objectively evaluate the performance of the proposed model in detecting varistor surface defects, we employ several widely recognized evaluation metrics. Specifically, we utilize Precision (*P*), Recall (*R*), Average Precision (AP), and mean Average Precision (mAP) as the primary performance indicators. Equations (5)–(8) define the mathematical formulations for these metrics:

(5) $$P=\frac{TP}{TP+FP}$$

Here, TP (True Positives) denotes the number of positive samples correctly predicted by the model, whereas FP (False Positives) denotes the number of negative samples incorrectly predicted as positive. Precision (*P*) measures the accuracy of the model’s predictions, defined as the proportion of true positives among all predicted positives. A higher *P* indicates that the detected results are more reliable.

(6) $$R=\frac{TP}{TP+FN}$$

In Equation (6), FN (False Negatives) denotes the number of positive samples incorrectly predicted as negative. Recall (*R*) reflects the model’s capability to retrieve true targets, defined as the proportion of correctly detected positives among all ground-truth positives. A higher *R* indicates fewer missed detections and stronger coverage of the target objects.

(7) $$AP=\int_{0}^{1}P(R)dR$$

(8) $$mAP=\frac{\sum_{i=1}^{n}AP_{i}}{n}$$

Average Precision (AP) is used to evaluate the detection performance for a single class. It is computed as the area under the Precision–Recall (PR) curve, thereby summarizing detection quality across different confidence thresholds. Furthermore, mean Average Precision (mAP) is obtained by averaging the AP values over all classes. In Equation (8), $AP_{i}$ represents the Average Precision of the i-th defect category, and n denotes the total number of categories. As an overall metric, mAP characterizes the comprehensive performance of an object detector and is one of the most important criteria for comparing detection algorithms.

Furthermore, we incorporate the number of parameters (Params) and Floating-Point Operations (FLOPs) as auxiliary metrics to assess the model’s efficiency. Params represents the total count of learnable parameters, serving as a measure of the model’s memory footprint and structural complexity. Meanwhile, FLOPs quantifies the number of floating-point operations required for a single forward inference pass, reflecting the computational load and runtime efficiency of the network.

### 4.3. Comparison of DAER-YOLO with Mainstream Object Detection Methods

To validate the effectiveness and superiority of the proposed DAER-YOLO model, we benchmarked it against several mainstream object detectors using the same dataset. The evaluation metrics include Precision (*P*), Recall (*R*), mean Average Precision (mAP@50, mAP@50:95), number of parameters (Params), computational complexity (GFLOPs), and inference speed (Frames Per Second, FPS). To ensure a fair and rigorous speed comparison, we conducted FPS testing under identical hardware conditions. Specifically, we employed FP16 half-precision for all lightweight models during inference. The only exception was the DINO model, which utilized FP32 precision due to its specific architectural constraints. Table 1 summarizes the comprehensive experimental results.

As shown in Table 1, DAER-YOLO maintains an overall comparable level to existing lightweight object detectors in terms of Precision and Recall, indicating its stable performance in defect detection capability and false positive control. More importantly, DAER-YOLO achieves the best results in the comprehensive evaluation metrics mAP@50 and mAP@50:95, reaching 95.5% and 64.4%, respectively. Compared to the YOLOv11n baseline, its mAP@50:95 is improved by 2.3%. Furthermore, compared to the SLF-YOLO [34] model designed for industrial defect detection, DAER-YOLO yields a 2.7% improvement in mAP@50:95, and a 7.6% improvement over the DAYOLOv3t [23] model, which is specifically tailored for varistor detection. The mAP@50:95 metric comprehensively reflects localization precision across various IoU thresholds. The substantial gains in this metric prove that DAER-YOLO maintains high detection quality even under strict IoU constraints. This demonstrates superior localization capabilities, particularly in challenging scenarios involving complex morphological defects such as edge damage and contour deformation.

Regarding lightweight characteristics and inference efficiency, DAER-YOLO has 3.02 M parameters, a computational complexity of 7.4 GFLOPs, and an inference speed of 57.05 FPS. Although the integrated defect perception and edge reconstruction modules decrease the inference speed compared to the baseline YOLOv11n (83.75 FPS), this trade-off results in an effective improvement in detection accuracy. Furthermore, the speed of 57.05 FPS still meets the 30 FPS standard typically required for real-time industrial inspection. Additionally, compared to similar industrial models, DAER-YOLO’s inference speed is higher than those of SLF-YOLO (54.58 FPS) and DAYOLOv3t (46.95 FPS), while maintaining fewer parameters and lower computational complexity. Compared with large-scale networks such as RT-DETR and DINO, the advantages of DAER-YOLO in computational overhead and real-time performance are more pronounced. Overall, while improving defect detection accuracy, DAER-YOLO maintains competitive lightweight characteristics and inference efficiency, providing a reliable algorithmic basis for deployment in environments with limited computational resources.

**Figure 9:**
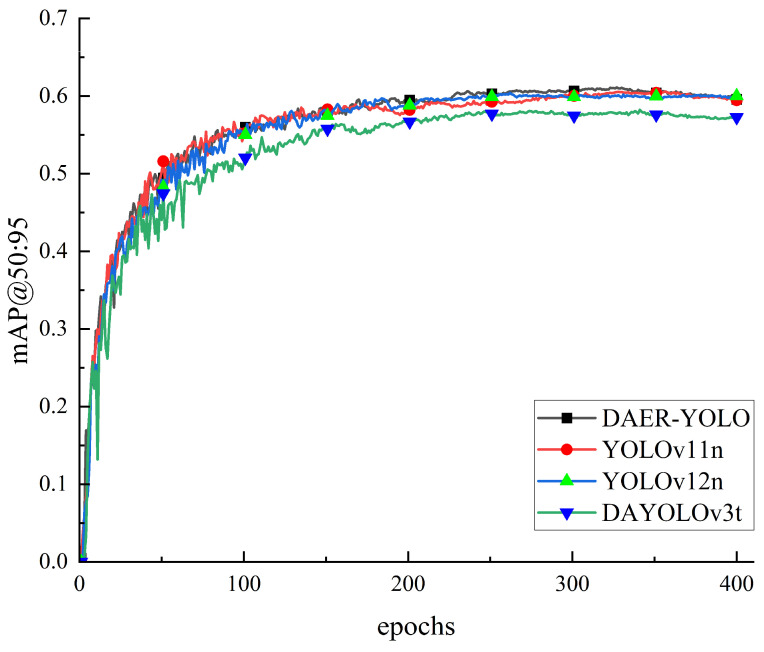
Comparison of mAP@0.5:0.95 training curves of DAER-YOLO and other YOLO-series object detection models.

To evaluate the convergence behavior and stability of different models during training, we compare the mAP@50:95 curves of DAER-YOLO, YOLOv11n, YOLOv12n, and DAYOLOv3t, as illustrated in Figure 9. During the initial training phase, all models exhibit a rapid upward trend in mAP@50:95. Notably, the improvement trajectory of DAER-YOLO is relatively continuous and smooth with fewer oscillations, suggesting steady gradient updates during the feature learning stage. As training progresses, DAER-YOLO gradually reaches a higher performance level and maintains a stable convergence state in the middle and later stages. In contrast, some of the comparison models experience varying degrees of fluctuation, reflecting a higher sensitivity to the training process when learning complex defect features. In summary, DAER-YOLO shows consistent convergence speed and training stability, which supports more reliable training for varistor defect detection.

**Figure 10:**
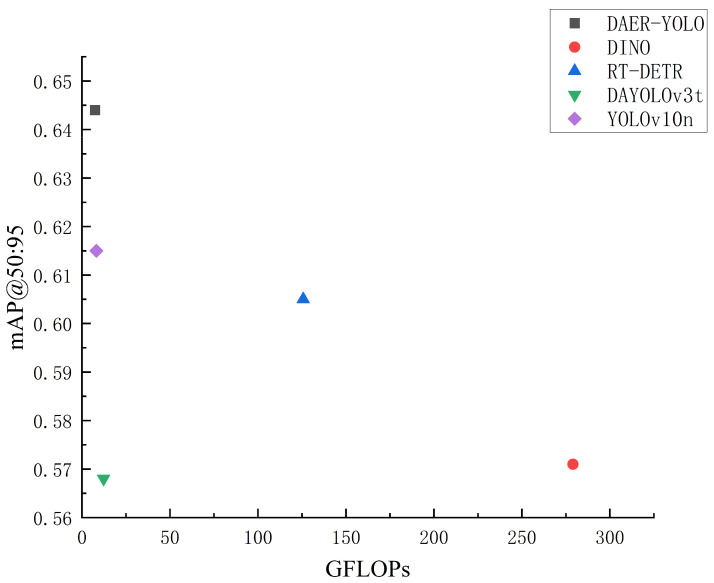
Performance comparison of DAER-YOLO and other mainstream object detection models in terms of GFLOPs and mAP@0.5:0.95.

Considering both detection accuracy and computational cost, we further analyze the performance of different models in terms of mAP@0.5:0.95 and GFLOPs, as illustrated in Figure 10. DAER-YOLO achieves a higher mAP@0.5:0.95 with a relatively low GFLOPs budget, positioning it on the Pareto frontier of the accuracy–efficiency trade-off. Compared to computation-intensive detectors such as DINO and RT-DETR, DAER-YOLO reduces the inference cost. Meanwhile, it provides higher detection accuracy than lightweight baselines such as DAYOLOv3t and YOLOv10n. These results indicate that DAER-YOLO improves feature representation and object localization while maintaining a lightweight design, achieving a balance between accuracy and efficiency for varistor surface defect detection.

### 4.4. Visual Comparison of Detection Results for DAER-YOLO

**Figure 11:**
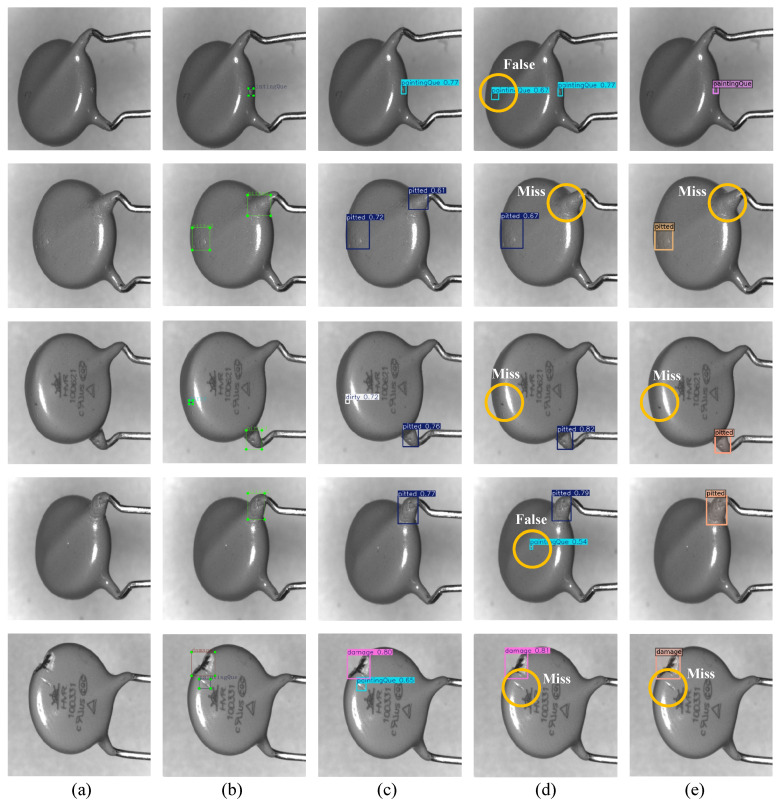
Visual comparison of DAER-YOLO with existing object detection methods. (**a**) Original image, (**b**) ground truth, (**c**) DAER-YOLO, (**d**) YOLOv11n, and (**e**) DINO.

To qualitatively evaluate the detection performance of DAER-YOLO, we conducted comparative analyses against a representative single-stage detector, YOLOv11n, and a two-stage detector, DINO [33]. Figure 11 displays the detection results of these three models on varistor images from the test set. As observed in the figure, the predicted bounding boxes generated by DAER-YOLO localize defect regions with fewer obvious false positives or missed detections. In contrast, YOLOv11n exhibits certain false positives and missed detections when identifying small targets and edge defects. Meanwhile, although DINO effectively avoids false positives, it still suffers from missed detections regarding small-to-medium targets and edge defects. These results suggest that the defect perception and edge reconstruction mechanisms enhance the representation of surface defect details. Under the specific conditions of the current custom industrial dataset, the detection accuracy and robustness of DAER-YOLO show targeted advantages over the baseline and comparison models.

### 4.5. Ablation Studies

To further validate the effectiveness of the proposed modifications, we conducted several ablation experiments under the same dataset settings with consistent training and testing protocols. Based on the YOLOv11n architecture, an improved network was developed by incorporating the C3k2-iEMA module into the neck and replacing the original upsampling operator. Four experimental configurations were established to evaluate the contribution of each component. Table 2 summarizes the ablation results.

As indicated by the results in Table 2, incorporating the C3k2-iEMA module into the YOLOv11n baseline model increases mAP@50 and mAP@50:95 by 1.7% and 1.4%, respectively. This suggests that the C3k2-iEMA module improves feature representation by better preserving small targets and fine-grained information during multi-scale feature fusion, thereby enhancing the model’s perception of tiny defects on varistors. When solely replacing the original upsampling operators with the EUCB module, mAP@50 and mAP@50:95 improve by 0.9% and 1.3%, respectively. This indicates that the EUCB module can more adequately recover spatial details during the feature reconstruction stage. It provides better reconstruction for edge damage and small-scale defects, helping to mitigate issues such as feature blurring and information loss.

By simultaneously integrating the C3k2-iEMA and EUCB modules, the model achieves an mAP@50:95 of 64.4%. Objectively, compared to introducing only the C3k2-iEMA module, the mAP@50 of the full model shows a marginal decrease of 0.1%. This phenomenon may stem from a feature optimization competition during the multi-task learning process: C3k2-iEMA focuses on utilizing multi-scale attention to capture low-frequency, diffuse, and weakly salient defect features, whereas EUCB is dedicated to the sharpening and spatial reconstruction of high-frequency physical edges. Their joint optimization induces slight fluctuations under lenient IoU thresholds; however, for mAP@50:95, which requires more precise bounding box overlap, the combined model shows a measurable improvement. These experimental results suggest that the C3k2-iEMA and EUCB modules provide synergistic benefits within DAER-YOLO. They jointly improve defect perception and edge localization from both the feature enhancement and reconstruction levels, resulting in improved overall performance for varistor surface defect detection.

To investigate the detection performance on specific morphological defects, we analyzed the Average Precision (AP) for surface contamination, edge breakage, and missing bottom across different ablation configurations. Table 3 presents the detailed results of this comparative analysis.

The data in Table 3 indicate that the C3k2-iEMA module improves the model’s detection performance for surface contamination and missing bottom defects. Specifically, the AP@50 for surface contamination increases from 76.8% to 81.6%, while for the missing bottom, it rises from 94.5% to 99.5%. This suggests that through its multi-scale perception and feature focusing capabilities, the C3k2-iEMA module can extract defect features from low-contrast and small regions, thereby enhancing the perception of weakly salient defects. However, when solely introducing this module, the performance for edge breakage shows a slight decline under the stricter AP@50:95 metric (from 67.5% to 66.5%). This reflects the limitations of relying on a single perception mechanism for the high-quality reconstruction of irregular broken edges. On the other hand, the independent introduction of the EUCB module leads to a distinct improvement in the edge breakage category; its AP@50 reaches 99.5%, and its AP@50:95 increases to 71.0%. These results verify that the EUCB module mitigates feature loss during upsampling through cross-channel interaction, supporting high-quality reconstruction for irregular broken edges.

When both modules are integrated into the final DAER-YOLO model, they exhibit synergistic effects. Specifically for the edge breakage category, the combined modules maintain the AP@50 at 99.5% while increasing the AP@50:95 to 76.0%, which represents an 8.5% improvement over the baseline model. Objectively, during the multi-task joint optimization process, a slight competition in feature optimization occurs between high-frequency edge reconstruction and low-frequency contamination feature extraction. Consequently, compared to solely using the C3k2-iEMA module, the AP@50 of the final model for surface contamination and missing bottom shows marginal fluctuations due to this trade-off. Nevertheless, the overall performance remains higher than that of the baseline model. These category-level ablation results demonstrate that C3k2-iEMA focuses on enhancing the detection of weakly salient targets, while EUCB optimizes the fit of edge contours to improve localization precision. Together, they form a complementary mechanism.

To verify the necessity of the internal components within the C3k2-iEMA module—specifically the EMA mechanism, the iRMB structure, and the Feed-Forward Network (FFN)—we conducted a fine-grained ablation study based on the YOLOv11n baseline. Table 4 presents the results of these experiments.

Table 4 indicates that introducing individual components involves distinct performance trade-offs. When solely introducing C3k2-EMA, the model increases mAP@50 to 94.6% with a computational cost of 6.1 GFLOPs; however, the Precision (P) and mAP@50:95 decrease compared to the baseline, suggesting the limitations of relying on a single attention mechanism for feature transformation. Conversely, although independently introducing C3k2-iRMB or C3k2-FFN improves the Recall (R) and mAP@50:95, the lack of attention guidance to filter background noise leads to a decline in Precision, reflecting a higher susceptibility to false positives. When integrated into the complete C3k2-iEMA module, the model shows synergistic effects. In the full configuration, the Precision (P) rebounds to 94.2%, while mAP@50 and mAP@50:95 reach 95.6% and 63.5%, respectively. Notably, compared to the single C3k2-iRMB, the complete C3k2-iEMA adds only 0.1 GFLOPs in computational overhead but addresses the false positive issue of the former. This result indicates that the complete module, through the complementary mechanisms of EMA suppressing background noise and iRMB enhancing edge features, achieves a balance between local feature perception and global semantic representation under lightweight constraints.

### 4.6. Model Interpretability and Reliability Analysis

To provide validation beyond standard evaluation metrics, this section analyzes the proposed DAER-YOLO model from two perspectives: feature heatmaps and performance stability across multiple random seed runs.

#### 4.6.1. Analysis of the Thermal Map Effect of the DAER-YOLO Model

**Figure 12:**
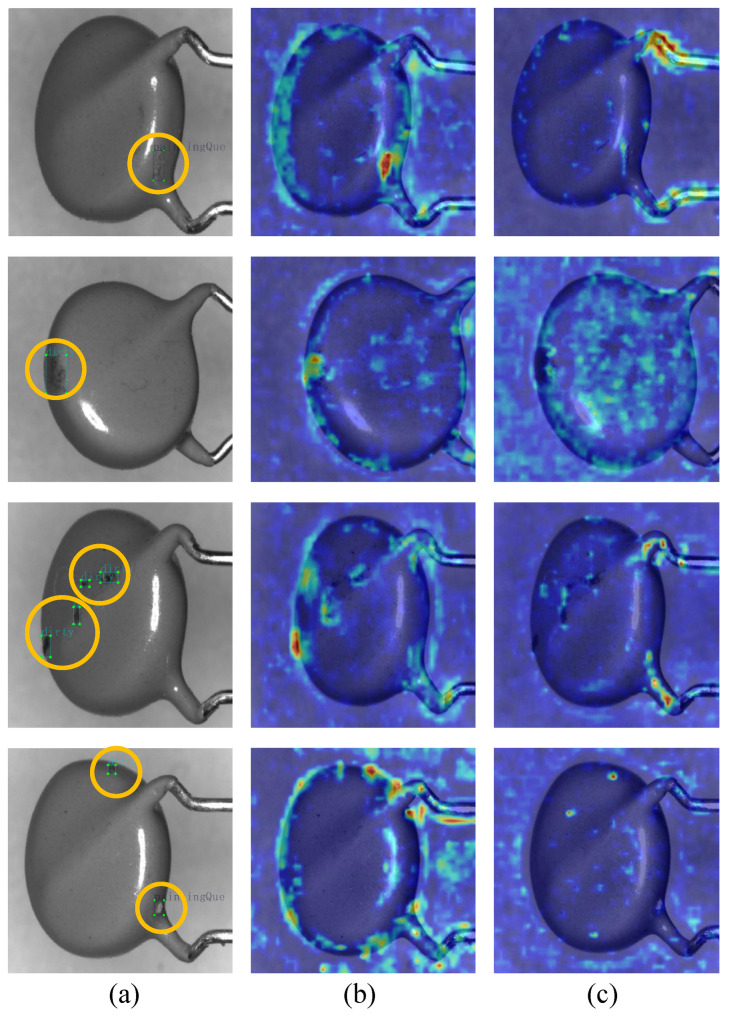
Heatmap comparison between the DAER-YOLO and YOLOv11n models. (**a**) Original images with defect annotations; (**b**) heatmaps of DAER-YOLO; (**c**) heatmaps of YOLOv11n.

To investigate the mechanisms of the proposed modules through the lens of network interpretability, we utilized Grad-CAM++ (Gradient-weighted Class Activation Mapping ++) [35] to visualize and analyze feature responses during inference. Grad-CAM++ generates pixel-level heatmaps by calculating the spatial gradient weights of target class feature maps, reflecting the regions the model prioritizes during detection. Figure 12 compares the feature response heatmaps of DAER-YOLO and the YOLOv11n baseline using test set images. Specifically, column (a) displays the original input images with ground truth (GT) bounding boxes; columns (b) and (c) show the heatmaps generated by DAER-YOLO and YOLOv11n, respectively.

For the single small target defect scenarios in the first and second rows of Figure 12, the high-response regions of the DAER-YOLO model cover the actual defect locations. Reflecting the influence of the EUCB module, the heatmaps of DAER-YOLO also show response bands along the edge regions of the varistors, suggesting the model retains and reconstructs edge contour features during inference. In contrast, the feature responses of the YOLOv11n baseline are relatively dispersed and fail to focus effectively on the actual defect regions, with some higher activations distributed across defect-free surfaces or the background.

For the multi-target small defect scenarios in the third and fourth rows of Figure 12, DAER-YOLO simultaneously forms high-response focal points across multiple scattered defect regions while maintaining attention along the physical edges of the device. This indicates that the C3k2-iEMA module, through its multi-scale feature fusion and attention mechanisms, improves the perception of local low-contrast and tiny targets, thereby reducing interference from irrelevant background information. Conversely, when handling multiple small targets, YOLOv11n exhibits lower activation levels that do not cover all defect regions within the field of view, and it shows weaker focus on edge contours. Based on this visual comparison, the feature response distribution of DAER-YOLO is more consistent with the practical requirements for varistor surface defect detection, supporting its effectiveness in small target perception and edge feature reconstruction.

#### 4.6.2. Statistical Reliability Analysis

To mitigate the impact of stochasticity during deep learning training and enhance the statistical reliability of the results, we conducted supplementary experiments using multiple random seeds (seed = 0, 1, and 42). Table 5 summarizes these results.

Table 5 shows that although typical performance fluctuations occur across different runs—such as the slight decrease in DAER-YOLO’s performance at seed = 42—the average performance of the proposed model consistently exceeds the YOLOv11n baseline. Specifically, DAER-YOLO achieves an average mAP@50 of 95.30% and an average mAP@50:95 of 64.27%, representing average gains of 0.93% and 1.87%, respectively, over the baseline. These results from multiple experimental runs indicate that the performance improvements are statistically consistent, supporting the reliability of our findings.

## 5. Discussion

For the varistor surface defect detection task, the proposed DAER-YOLO model achieves competitive accuracy. The performance improvements are primarily attributed to the synergistic design of feature representation enhancement and feature reconstruction optimization. Furthermore, this work provides a practical technical reference for developing lightweight detection models for other industrial components where small target defects and irregular breakages coexist.

From the perspective of feature representation, the C3k2-iEMA module enhances interactions across multi-scale features, facilitating a more thorough integration of low-level spatial cues and high-level semantic information. This multi-scale optimization helps mitigate the attenuation of small-defect signals during progressive feature propagation, thereby improving the perception of fine-grained defects. Additionally, combining EMA with the inverted residual structure (iRMB) enables the network to selectively attend to low-contrast regions. This partially suppresses background noise and specular reflections, which improves the discriminability of defective areas. Regarding feature reconstruction, the EUCB upsampling mechanism improves the recovery of boundary and detail information while maintaining computational efficiency. Compared with conventional upsampling schemes, EUCB reduces the edge blurring typically caused by excessive feature smoothing. This results in clearer representations of defects—such as edge damage and contour deformation—within high-resolution feature maps, ultimately facilitating more accurate localization and category discrimination by the detection head.

Although DAER-YOLO demonstrates effective overall performance in the experiments, it has certain limitations.

First, this research relies primarily on a grayscale image dataset, lacking systematic validation under color imaging conditions. While using grayscale images reduces model complexity, it inevitably omits certain color and texture information. This omission may restrict the model’s adaptability in more complex industrial lighting environments or multimodal imaging scenarios.

Second, highly irregular defect morphologies on varistor surfaces remain difficult to categorize clearly within the existing annotation framework. Consequently, the model lacks sufficient class prior information during training, which can affect its recognition performance on these anomalous samples.

Third, regarding generalization capability, we validated the model primarily on a custom varistor dataset acquired in a specific environment. Industrial manufacturing scenarios are complex; defect morphologies and background noise often exhibit significant discrepancies across different batches, production lines, or component types. Nevertheless, the core mechanisms of DAER-YOLO show broader application potential. The C3k2-iEMA module can adapt to detect low-contrast defects on other components, and the EUCB module can transfer to visual tasks requiring contour boundary preservation. However, because the current model is optimized specifically for grayscale varistor images, its direct generalization to color surface detection or other unseen domains remains limited without fine-tuning or transfer learning for feature calibration. Future research will introduce broader public datasets to validate its cross-domain robustness.

In summary, by jointly optimizing feature representation and feature reconstruction, DAER-YOLO achieves an effective balance between detection accuracy and computational efficiency for varistor surface defect inspection, demonstrating its potential for practical engineering applications. Furthermore, the identified limitations provide clear directions for future research.

## 6. Conclusions

Varistor surface defect detection faces critical challenges, such as identifying small targets, modeling complex defects, and preserving edge features. To address these, we propose DAER-YOLO, a lightweight model integrating defect perception enhancement and edge reconstruction mechanisms.

At the feature representation level, the model incorporates the C3k2-iEMA module into the neck network. It integrates the EMA mechanism, the iRMB structure, and an FFN to facilitate cross-scale feature interaction. This strengthens the representation of small and irregular defects, improving accurate localization. At the feature reconstruction level, the Efficient Up-Convolution Block (EUCB) replaces traditional upsampling operators. While maintaining computational efficiency, it improves the recovery of edge and detail information and reduces the impact of specular reflections on feature extraction, helping to mitigate false positives and missed detections. Ablation studies indicate that C3k2-iEMA and EUCB provide both independent gains and synergistic effects, jointly improving the overall detection performance.

In experimental evaluations, DAER-YOLO achieves 95.5% on mAP@50 and 64.4% on mAP@50:95, with both Precision and Recall reaching 91.8%. While maintaining comparable parameters and computational complexity, its performance outperforms representative models such as DAYOLOv3t, YOLOv12n, and DINO, showing a balance between accuracy and efficiency. Furthermore, visualization results indicate that DAER-YOLO accurately localizes and identifies defect regions even in grayscale images with specular reflections, indicating its potential for practical application in specific industrial inspection scenarios.

Future research will focus on improving data quality and system-level implementation to further enhance the utility and robustness of varistor surface defect detection. Specifically, we will develop a high-quality color image dataset covering diverse defect types and imaging conditions, and investigate detection methods tailored for color features. Concurrently, we will develop a real-time detection system integrating industrial cameras, deep learning inference models, and host computer software to achieve online recognition and precise localization. Furthermore, we will introduce a simulated cutting process based on the detection results, providing technical support for the intelligent quality sorting and automated production of varistors.

## Figures and Tables

**Table 1:** Comparison results of DAER-YOLO and other mainstream object detection models on the test set.

Model	*P*%	*R*%	mAP @50 (%)	mAP @50:95 (%)	Params/M	GFLOPs	FPS
YOLOv11n [11]	92.3	90.1	93.9	62.1	2.58	6.3	83.75
YOLOv8n [28]	92.4	92.7	94	61.5	2.68	6.8	64.43
YOLOv9t [29]	94.2	90.1	94.8	62.5	1.73	6.4	29.17
YOLOv10n [30]	87.7	82.4	92.1	61.5	2.69	8.2	39.43
YOLOv12n [31]	92.8	90.2	95.3	62	2.56	6.3	42.73
DAYOLOv3t [23]	89.5	81.8	89.4	56.8	6.9	12.3	46.95
RT-DETR [32]	90.3	89.7	91.8	60.5	41.95	125.7	12.11
DINO [33]	-	-	91.8	57.1	46.60	279	4.50
SLF-YOLO [34]	91	89.8	93.7	61.7	9.65	24.6	54.58
**DAER-YOLO**	91.8	91.8	**95.5**	**64.4**	3.02	7.4	57.05

**Table 2:** Ablation results of DAER-YOLO on the test set, where a check mark (√) indicates whether the corresponding module is enabled.

YOLOv11n	C3k2-iEMA	EUCB	mAP@50 (%)	mAP@50:95 (%)	Params/M	GFLOPs
√			93.9	62.1	2.58	6.3
√	√		**95.6**	63.5	2.93	6.9
√		√	94.8	63.4	2.67	6.8
√	√	√	95.5	**64.4**	3.02	7.4

**Table 3:** Ablation study on the detection accuracy of specific varistor defect categories. A check mark (√) indicates that the specified module is enabled.

YOLOv11n	C3k2-iEMA	EUCB	Surface Contamination		Edge Damage		Missing Bottom	
			AP@50	AP@50:95	AP@50	AP@50:95	AP@50	AP@50:95
√			76.8	47.1	95.5	67.5	94.5	55.9
√	√		81.6	48.2	98.2	66.5	99.5	58.7
√		√	78	45.4	99.5	71	94.2	55.6
√	√	√	80.4	48.1	99.5	76	96.9	57.9

**Table 4:** Results of the fine-grained ablation study on the internal components of the C3k2-iEMA module. The √ symbol indicates that the corresponding module is used in the baseline model.

YOLOv11n	+EMA	+iRMB	+FFN	C3k2-iEMA	*P* (%)	*R* (%)	mAP @50 (%)	mAP @50:95 (%)	Params/M	GFLOPs
√					92.3	90.1	93.9	62.1	2.58	6.3
√	√				91.3	91.3	94.6	60.7	2.49	6.1
√		√			89.2	93.2	94.0	63.0	2.93	6.8
√			√		87.6	91.6	94.6	62.4	2.84	6.7
√				√	94.2	90.1	95.6	63.5	2.93	6.9

**Table 5:** Statistical reliability analysis across different random seeds.

Model	Seed	P	R	mAP@50 (%)	mAP@50:95 (%)
YOLOv11n	0	92.3	90.1	93.9	62.1
	1	92.1	92.7	94.7	62.4
	42	92.8	89.3	94.5	62.7
	Mean ± Std	92.40 ± 0.36	90.70 ± 1.78	94.37 ± 0.42	62.40 ± 0.30
DAER-YOLO	0	91.8	91.8	95.5	64.4
	1	95.3	90.8	95.8	64.9
	42	91.9	90.0	94.6	63.5
	Mean ± Std	**93.00 ± 1.99**	**90.87 ± 0.90**	**95.30 ± 0.62**	**64.27 ± 0.71**

## Data Availability

The original contributions presented in this study are included in the article. Further inquiries can be directed to the corresponding authors.
